# Protective effects of systemic dermatan sulfate treatment in a preclinical model of radiation-induced oral mucositis

**DOI:** 10.1007/s00066-018-1280-8

**Published:** 2018-03-01

**Authors:** Sylvia Gruber, Katharina Frings, Peter Kuess, Wolfgang Dörr

**Affiliations:** 1 Christian Doppler Laboratory for Medical Radiation Research for Radiation Oncology, Medical University of Vienna, Vienna, Austria; 2 Department of Radiation Oncology, Applied and Translational Radiobiology (ATRAB), Medical University of Vienna, Währinger Gürtel 18–20, 1090 Vienna, Austria; 3 Platform Radiooncology and Nuclear Medicine, Department for Companion Animals and Horses, University of Veterinary Medicine of Vienna, Vienna, Austria

**Keywords:** Radiotherapy, Oral mucositis, Dermatan sulfate, Mouse model, Radiotherapie, Orale Mukositis, Dermatansulfat, Mausmodell

## Abstract

**Purpose:**

Oral mucositis is a frequent, dose-limiting side effect of radio(chemo)therapy of head-and-neck malignancies. The epithelial radiation response is based on multiple tissue changes, which could offer targets for a biologically tailored treatment. The potential of dermatan sulfate (DS) to modulate radiation-induced oral mucositis was tested in an established preclinical mucositis model.

**Methods:**

Irradiation was either applied alone or in combination with daily DS treatment (4 mg/kg, subcutaneously) over varying time intervals. Irradiation comprised single dose irradiation with graded doses to the lower tongue surface or daily fractionated irradiation of the whole tongue. Fractionation protocols (5 × 3 Gy/week) over one (days 0–4) or two weeks (days 0–4, 7–11) were terminated by an additional local single dose irradiation to a defined treatment field on the lower tongue surface to induce the mucosal radiation response. The additional single dose irradiation (top-up) on day 7 (after one week of fractionation) or day 14 (after 2 weeks of fractionation) comprised graded doses in order to generate full dose–effect curves. Ulceration of the epithelium of the lower tongue, corresponding to confluent mucositis, was analysed as clinically relevant endpoint. Additionally, the time course parameters, latent time and ulcer duration were analysed.

**Results:**

DS treatment significantly reduced the incidence of ulcerations. DS application over longer time intervals resulted in a more pronounced reduction of ulcer frequency, increased latent times and reduced ulcer duration.

**Conclusion:**

DS has a significant mucositis-ameliorating activity with pronounced effects on mucositis frequency as well as on time course parameters.

## Zusammenfassung

**Hintergrund und Ziel:**

Die orale Mukositis ist eine häufige, dosislimitierende Nebenwirkung der Radio(chemo)therapie von Kopf-Hals-Malignomen. Die epitheliale Strahlenreaktion basiert auf multiplen Gewebereaktionen, die Ziele biologisch angepasster Behandlungen darstellen können. Das Potenzial von Dermatansulfat (DS), die radiogene orale Mukositis zu modulieren, wurde in einem etablierten präklinischen Mukositis-Modell getestet.

**Methoden:**

Die Bestrahlung erfolgte entweder alleinig oder in Kombination mit täglicher DS-Behandlung (4 mg/kg, subkutan) über variierende Zeitintervalle. Die Bestrahlungsprotokolle umfassten alleinige Einzeldosen appliziert auf die Zungenunterseiten oder eine täglich fraktionierte Bestrahlung der gesamten Zunge. Die Fraktionierung (5 × 3 Gy/Woche) über eine (Tage 0–4) oder zwei Wochen (Tage 0–4, 7–11) wurde mit einer zusätzlichen lokalen Einzeldosisbestrahlung auf ein definiertes Bestrahlungsfeld auf der Zungenunterseite abgeschlossen, um die Strahlenreaktion der Schleimhaut zu induzieren. Die zusätzliche Einzeldosisbestrahlung (top-up) an Tag 7 (nach einwöchiger Fraktionierung) oder 14 (nach zweiwöchiger Fraktionierung) umfasste gestaffelte Dosen, um volle Dosis-Effekt-Kurven zu generieren. Ulzerationen des Epithels der Zungenunterseite, entsprechend einer konfluenten Mukositis, wurden als klinisch relevanter Endpunkt gewertet. Zusätzlich wurden die Parameter des zeitlichen Verlaufs, Latenzzeit sowie Ulkusdauer analysiert.

**Ergebnisse:**

Die DS-Behandlung reduzierte die Ulzerationsinzidenz signifikant. Eine DS-Applikation über längere Zeiträume resultierte in einer stärkeren Reduktion von Ulzerationsfrequenz sowie längeren Latenzzeiten und kürzeren Manifestationszeiten.

**Schlussfolgerung:**

DS zeigte eine signifikante mukositisreduzierende Aktivität, mit ausgeprägten Effekten auf Mukositisfrequenz und zeitlichen Verlauf.

## Introduction

Radiotherapy is a highly effective treatment option for head-and-neck cancers, but is limited by normal tissue adverse effects, as various critical organs at risk are usually located either within the planning target volume (PTV) or in the beam portals. Oral mucositis is the most frequent, dose-limiting early side effect of head-and-neck cancer radiotherapy, experienced by the majority of patients [1, 2]. Severe, confluent oral mucositis interferes with speaking and swallowing function, significantly impairs the patient’s quality of life and can require hospitalization and treatment interruptions [3, 4]. The overall treatment time is a crucial factor for the treatment outcome. Severe oral mucositis can necessitate unplanned treatment interruptions and thus directly compromises tumour control probability [5, 6]. Current prophylactic or management interventions are purely symptomatic and focus on pain management, improvement of oral hygiene and antibiosis [7, 8]. Although a variety of biology-based treatment strategies have been tested preclinically and in few initial clinical trials [9–11], so far none achieved clinical implementation. Oral mucositis, eventually manifesting as ulcerative lesions, is predominantly based on the inhibition of the epithelial proliferative capacity in the germinal tissue compartments, in face of ongoing superficial cell loss due to mechanical and/or chemical stress [12, 13]. The epithelial radiation response is regularly accompanied by inflammatory changes [14–16], and an early onset of local hypoxic conditions, as shown in preclinical studies [17]. The development of biology-based strategies needs to be based on the targeting of radiation-altered cellular signalling.

Dermatan sulfate (DS) belongs to the family of glycosaminoglycans and exerts pleiotropic biological functions, influencing the maintenance of tissue architecture, cellular adhesion, proliferation, coagulation, wound healing and inflammation [18–22]. DS activity targets multiple pathological (radiation-induced) tissue changes simultaneously and therefore was chosen for evaluation of its mucoprotective potential in the established mouse tongue irradiation model. So far, no radioprotective potential of DS has been reported in literature. In the present study, the effect of systemic DS treatment over varying time intervals in combination with single dose and fractionated irradiation on the induction of oral mucositis in the well-accepted mouse tongue irradiation model is assessed. The incidence of mucosal ulcerations, corresponding to confluent oral mucositis, was analysed as the clinically relevant endpoint. No comparable studies are found in the literature.

## Methods

All experiments were performed according to the current animal welfare legislation with approval by the respective Austrian authorities (file no. BMWF 66.009/0039-II/3b/2014).

### Animals and housing

For all experiments, mice of the inbred C3H/Neu strain from the breeding colony of the Department of Biomedical Research, MedUni Vienna, were used. Mice of both genders were included in the experiments, since earlier studies did not show any gender-related effects on the mucosal radiation response [23]. Mice were housed under controlled conditions of temperature (22 ± 2 °C) and humidity (55 ± 10%), and a day/night light rhythm of 12 h. The animals were kept in Makrolon® cages (1284L Eurostandard Type II L; Techniplast GmbH, Hohenpeißenberg, Germany), with a maximum of 5 animals per cage, on aspen wood bedding (ABEDD-LAB & VET Service GmbH, Vienna, Austria) and had free access to standard maintenance diet (ssniff Spezialdiäten GmbH, Soest, Germany) and fresh water ad libitum from standard perspex drinking bottles. The age of the mice at the onset of the experiments ranged from 8 to 12 weeks.

### Experimental design

For all irradiation procedures, a YXLON Maxishot X‑ray unit (Yxlon International X‑ray GmbH, Hamburg, Germany) was used. Dosimetric commissioning was performed for all used set-ups. The applied dose to the mice was measured using dedicated in-house build cylindrical polymethylmethacrylat (PMMA) phantoms simulating a mouse body. These phantoms had bores to insert ionization chambers and subsequently measure the dose at the point of interest. A detailed investigation was performed beforehand to consider the small beam geometry [24]. Standard dosimetric quality assurance was done regularly and the dose-rate was found to be constant. Adjustment of the irradiation time thus was applied to define the delivered dose. Irradiation techniques have been described in detail elsewhere [23, 25] and will only be briefly summarized subsequently.

**Fig. 1:**
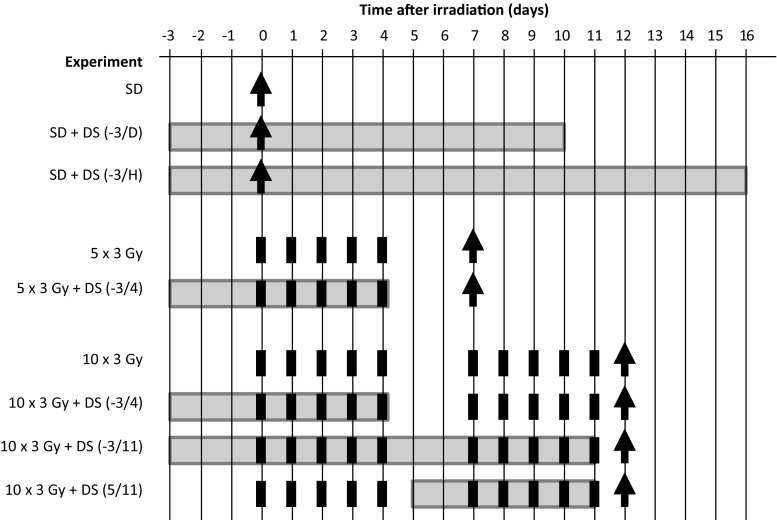
Experimental protocols. Irradiation protocols comprised single dose irradiation (SD, *arrow*) or daily fractionated irradiation with 3 Gy per day (*black square*) over one (5 × 3 Gy) or two weeks (10 × 3 Gy), followed by graded top-up doses (SD, 5 dose groups, 10 animals each) on day 7 or 14, respectively. Irradiation was either applied alone or in combination with daily 4 mg/kg dermatan sulfate (DS) treatment over varying time intervals, illustrated by the shaded area

An overview about the individual experimental protocols is given in Fig. 1.

### Single dose irradiation

**Fig. 2:**
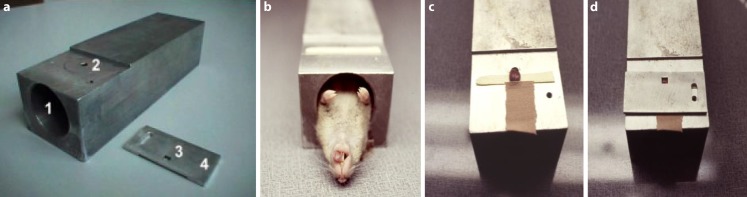
Single dose irradiation setup. For single dose irradiation, a customized aluminium block holder (**a**) was used. Mice were anaesthetized and placed in the central bore (**a***1*) in a supine position (**b**). The mouse tongue was gently pulled through the hole on the upper side of the block (**a***2*, **c**) and fixed with adhesive tape; the lower tongue surface facing the beam. A 3 × 3 mm^2^ opening (**a***3*) in the 1 mm thick aluminium plate (**a***4*), which was positioned centrally over the lower tongue surface, defined the treatment field (**d**). The aluminium setup shielded everything except the treatment field

Single dose irradiation was applied with graded doses (5 dose groups, 10 animals each) on day 0 to a limited treatment field of 3 × 3 mm^2^ of the lower tongue surface. In order to generate full dose–effect curves, graded doses comprised 7, 10, 12, 14 and 17 Gy. For this, anaesthetized mice (pentobarbitone sodium, 60 mg/kg intraperitoneally) were placed in the central bore (diameter 25 mm) of a prewarmed (ca. 35 °C) aluminum block in a supine position. The tongue of the mouse was gently pulled through a hole of 3 mm diameter in the roof of the block by means of forceps. The upper tongue surface was fixed to the block surface with adhesive tape. A 1 mm thick aluminum plate with a 3 × 3 mm^2^ window was positioned centrally over the lower tongue surface, thus defining the treatment field and shielding tip, margins and base of the tongue. The aluminium block with the mouse was positioned in a standardized way in the central beam, see Fig. 2.

The X‑ray unit was operated with a tube voltage of 25 kV with a current of 20 mA and a focus size of 5.5 mm. The beam is inherently filtered by 3 mm Be. For tongue irradiation, an additional 0.3 mm Al beam filter was installed, resulting in a dose rate of ca. 4 Gy/min at a focus-to-surface distance of 15 cm. The beam direction was vertical.

### Fractionated irradiation

**Fig. 3:**
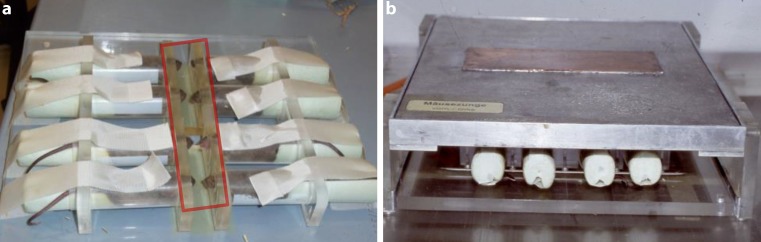
Fractionated irradiation setup. Fractionated irradiation was given to 8 mice simultaneously, no anaesthesia was required. Animals were positioned in the set-up of two rows of opposing plastic tubes with an inner diameter of 2 cm. The snouts were guided through conical holes into the irradiation field (*red rectangle*). Withdrawal of the animals was prevented by closing the rear ends of the plastic tubes with polystyrene wedges (**a**). The treatment field encompassed the whole snouts, including the tongues. The rest of the bodies were shielded (**b**)

Fractionated irradiation, starting on day 0, was given to the whole snouts of eight animals simultaneously. Un-anaesthetized animals were guided into a set-up of 2 rows of 4 opposing plastic tubes (inner diameter 2 cm). The snouts were positioned in conical holes (10 mm → 6 mm) of a perspex block at the front end of the tubes. The rear ends were closed to prevent withdrawal of the animals. The bodies of the mice were shielded caudally from a plane from the eyes to the throat with 12 mm of the Pb–Bi–Sn alloy MCP-96. The treatment volume thus included the snouts with the entire tongue, which was irradiated percutaneously with 3 Gy per fraction. The set-up was positioned in a standardized way in the central beam of the irradiation device. The setup for fractionated irradiation is illustrated in Fig. 3.

Fractionated irradiation, given either over one (days 0–4) or two weeks (days 0–4, 7–11) was concluded with graded local test (top-up) doses, on days 7 or 14, respectively, in analogy to single dose irradiation. Fractionated irradiation alone over one or two weeks does not lead to the development of mucositis, due to the repopulation process counteracting dose from the second treatment week onwards [26]. Therefore, the additional top-up irradiation was applied in order to induce mucositis. Initially, graded top-up irradiation after one week of fractionation comprised 5, 8, 10, 12 and 15 Gy. Due to the high number of responders in the lower dose groups, no ideal S‑shaped dose–response curve could be achieved and the doses were adapted for one week of fractionated irradiation in combination with DS treatment to 3, 6, 8, 10 and 13 Gy. Top-up irradiation after two weeks of fractionation comprised 5, 8, 10, 12 and 15 Gy. The same top-up doses were applied after additional DS treatment from day −3 to day 4 and day −3 to day 11. After the mucoprotective effect of DS became evident during the aforementioned experiments, the top-up doses were escalated to 9, 12, 14, 16 and 19 Gy for the experiment of two weeks of fractionation plus DS treatment from day 5 to day 11 to ensure full dose–response curves.

For fractionated irradiation, the YXLON Maxishot device was operated at 200 kV with a tube current of 20 mA and a focus size of 5.5 mm. For snout irradiation, an additional 4 mm Al and 0.6 mm Cu beam filter was used, which resulted in a dose rate of ca. 1 Gy/min at the focus-to-skin distance of 45.5 cm. The dose homogeneity between the individual snout positions was 3.2 ± 0.5%. The beam direction was vertical.

### Dermatan sulfate (DS)

A daily dose of 4 mg/kg dermatan sulfate (Sigma Aldrich, MO, USA, catalog no. C3788), dissolved in saline at a concentration of 1 mg/ml, was administered subcutaneously over varying time intervals. On irradiation days, the drug was administered 2 h after irradiation. On irradiation-free days, DS was administered between 11:00 am and 12:00 am, similarly to the treatment time on irradiation days and 24 h after the previous DS administration.

With single dose irradiation, DS was administered already 3 days prior to irradiation, from day −3 until diagnosis of ulceration (−3/D) or until healing of manifest ulcers was completed (−3/H). In animals, which did not develop ulcerations DS treatment was terminated when the ulcerations of the responders had healed.

In combination with fractionated irradiation over one week, DS treatment started 3 days prior the first fraction and was continued over the week of fractionation. DS was administered from day −3 until day 4 (−3/4), no DS was given over the irradiation-free weekend on days 5 and 6 or on the day of the local top-up irradiation, day 7.

With fractionated irradiation over two weeks, DS was applied over three different intervals.

#### DS administration protocol 1

DS treatment was started 3 days prior the first fraction (day 0) and continued over the first week of radiotherapy until day 4 (−3/4). No further DS treatment was given, before the experiment was concluded with the local top-up irradiation on day 14. No drug treatment was performed on the day of the local top-up irradiation.

#### DS administration protocol 2

DS was given over both weeks from day −3 until day 11 (−3/11). DS administration continued over the first irradiation-free weekend (days 5 and 6) and was terminated on day 11. DS was neither administered on the second irradiation-free weekend (days 12 and 13), nor on the day of the concluding top-up irradiation (day 14).

#### DS administration protocol 3

DS treatment was limited to day 5 until day 11 (5/11). DS was not administered during the first week of fractionation (days 0–4) and terminated after day 11. No DS was given on the second irradiation-free weekend (days 12 and 13) or on the day of the local top-up irradiation (day 14).

### Follow-up, endpoints and statistical analyses

Tongues were scored daily from the onset of first symptoms until complete re-epithelialization. For this, mice were immobilized with pentobarbitone sodium (ca. 40 mg/kg intraperitoneally). Mucosal ulceration, corresponding to confluent mucositis RTOG/EORTC grade 3, was used as the quantal, clinically relevant primary endpoint. Time course parameters, i. e. latent time from local single-dose/top-up irradiation to first ulcer diagnosis, and ulcer duration from first diagnosis to clinical healing, were analysed as secondary endpoints.

Python 3.6 programming language (Python Software Foundation, https://www.python.org/) was used for statistical procedures (StatsModels package) and illustration (Matplotlib toolkit). Logit analyses were performed, assuming a log-normal distribution without a threshold dose, in order to generate dose–effect curves. The latter are described by ED_50_ values (doses, at which ulceration is expected in 50% of the animals) and their standard deviation *σ*, and by *p*_dose_ values for the effect of dose on ulcer incidence. For the comparison of dose–effect relationships, a likelihood-ratio test was used, based on the logit model, resulting in *p*_vs. control_ values. Dose modification factors (DMF) describe the ratio of the ED_50_ values in the individual DS protocols to the control experiment without DS.

$$\text{DMF} = \frac{\text{ED}_{50} \text{ in presence of DS}}{\text{ED}_{50} \text{ in absence of DS}}$$

Mean latencies and ulcer durations were calculated from all responders in the respective experiment, independent of dose. For the comparison of time course parameters between experimental groups, the Student’s t‑test was used. A *p*-value ≤0.05 was regarded statistically significant.

## Results

Irradiation and DS treatment were well tolerated. No reactions other than the mucosal radiation response were observed. Neither irradiation nor DS treatment negatively influenced bodyweight, behaviour or appearance of the animals. Results are summarized in Table 1.

**Table 1:** The effect of systemic DS treatment on oral mucositis (mouse)

Number of fractions	Administration interval (days)	ED_50_ ± σ (Gy)^a^	*p*_dose_^b^	*p* vs. Control^c^	DMF	Latent time ± SD (days)^d^	*p* vs. Control	Ulcer duration ± SD (days)^d^	*p* vs. Control
*Single dose irradiation*									
–	–	11.9 ± 1.2	<0.001	–	–	11.8 ± 0.9	–	3.1 ± 0.4	–
–	−3–D	14.1 ± 0.1	<0.001	**0.002**	1.1	12.4 ± 0.8	0.076	2.6 ± 0.8	0.108
–	−3–H	14.0 ± 1.0	0.001	**0.003**	1.2	12.2 ± 0.8	0.885	2.7 ± 0.7	0.215
*Fractionation 1 week*									
5	–	6.9 ± 3.2	0.006	–	–	8.2 ± 0.8	–	3.3 ± 0.6	–
5	−7	12.3 ± 0.9	0.005	**<0.001**	1.8	8.9 ± 0.7	0.060	2.0 ± 0.6	**<0.001**
*Fractionation 2 weeks*									
10	–	8.3 ± 2.1	0.001	–	–	8.1 ± 0.9	–	3.4 ± 0.8	–
10	−3–4	10.4 ± 1.4	<0.001	0.055	1.2	8.9 ± 0.5	**0.008**	2.9 ± 0.5	0.150
10	−3–11	15.4 ± 1.3	0.024	**<0.001**	1.8	9.2 ± 0.5	**<0.001**	2.2 ± 0.5	**0.027**
10	5–11	12.6 ± 1.4	<0.001	**0.001**	1.5	9.7 ± 0.6	**<0.001**	2.1 ± 0.6	**0.002**

*DS* dermatan sulfate, *D* diagnosis, *H* healing, *ED* effective dose, *DMF* dose modifying factor, *SD* standard deviation

^a^Standard deviation σ of the ED_50_ value, resulting from logit analyses

^b^p-values for the radiation dose dependence of ulcer incidence, resulting from logit analyses

^c^p-values for the difference between dose–effect curves, resulting from maximum likelihood analyses

^d^Relative to the day of local irradiation, i. e. in fractionation protocols relative to the day of top-up irradiation

### Primary endpoint—the effect of DS on the dose response of mucositis incidence

#### Single dose irradiation

**Fig. 4:**
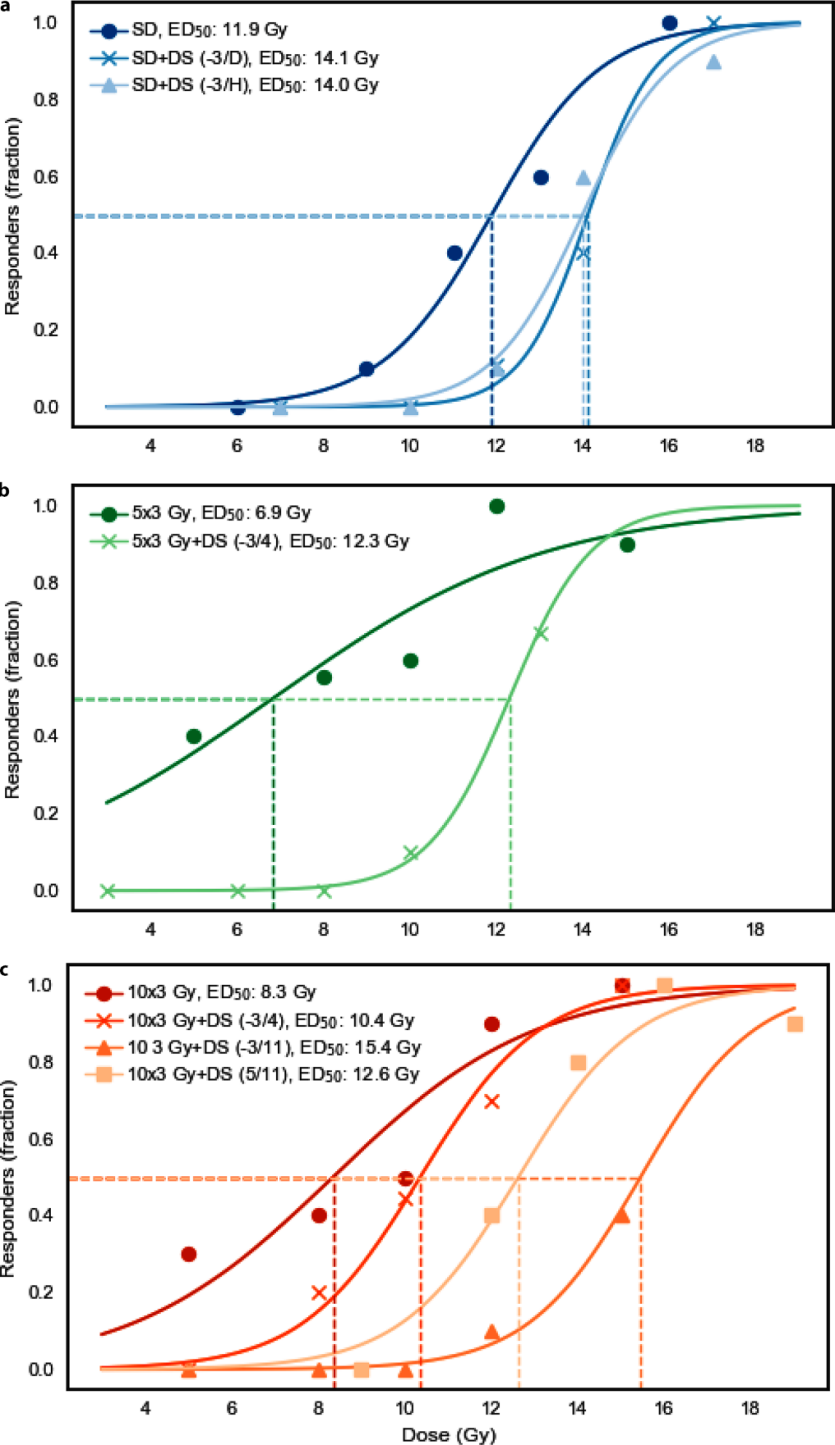
Effect of systemic dermatan sulfate (DS) treatment on radiation-induced mucositis on the lower tongue surface. Dose–responses curves for mucositis induction (responder = animal with mucositis) after irradiation ± DS treatment. Irradiation protocols comprised single dose irradiation or daily fractionated irradiation over one (days 0–4) or two weeks (0–4, 7–11), followed by graded top-up doses (5 dose groups, 10 animals each) on day 7 or 14, respectively. ED_50_ values (iso-effective doses to induce mucositis in 50% of animals, *dotted lines*) result from logit analyses. Systemic DS administration resulted in significantly increased ED_50_ values in all protocols tested, except when the treatment time was limited the first week of two weeks of fractionation. The corresponding *p*-values of maximum likelihood analyses are given in Table 1

Single dose irradiation alone resulted in an ED_50_ of 11.9 ± 1.0 Gy. A highly significant dose dependence was found for the ulcer incidence (*p* = 0.0005). Additional DS treatment significantly reduced the ulcer incidence in both single dose irradiation protocols tested and shifted the responder probability curve, derived from logit analyses, towards higher doses. Systemic DS treatment increased the ED_50_ to 14.1 ± 0.1 Gy (*p* = 0.002) and 14.0 ± 1.0 Gy (*p* = 0.003), compared to the ED_50_ of single dose irradiation alone, when administered from day −3 until ulcer diagnosis or ulcer healing, respectively (Fig. 4a).

#### Fractionated irradiation

Fractionated irradiation over one week yielded an ED_50_ for test irradiation of 6.9 ± 3.2 Gy. Additional DS treatment from day −3 until day 4 increased the ED_50_ of the concluding top-up irradiation to 12.3 ± 0.9 Gy (*p* < 0.001; Fig. 4b).

For fractionated irradiation over two weeks alone, an ED_50_ of 8.3 ± 2.1 Gy was calculated. Additional DS treatment shifted the responder probability curve towards higher doses in all three DS treatment schedules tested, with the longest treatment time (day −3 until day 11) achieving the most pronounced effect. DS application over the first week, from day −3 until day 4, the ED_50_ increased to 10.4 ± 1.4 Gy (*p* = 0.055), as compared to two weeks of fractionation alone. DS treatment from day −3 until day 11, gave an ED_50_ of 15.4 ± 1.3 Gy (*p* < 0.001). When administered in the second week of fractionated irradiation only, DS increased the ED_50_ to 12.6 ± 1.4 Gy (*p* = 0.00; Fig. 4c).

### Secondary endpoints—the effect of DS on the time course parameters

#### Single dose irradiation

After single dose irradiation, the mean latent time (±SD) to ulcer diagnosis was 11.8 ± 0.9 days. On average, ulcerations lasted for 3.1 ± 0.4 days. Additional DS treatment from day −3 until diagnosis and healing, respectively, delayed the mean time to manifestation of ulcerations to 12.4 ± 0.8 days (*p* = 0.076) and 12.2 ± 0.8 days (*p* = 0.885). Ulcer duration was reduced to 2.6 ± 0.8 days (*p* = 0.108) and 2.7 ± 0.7 days (*p* = 0.215), respectively (Fig. 6).

#### Fractionated irradiation

**Fig. 5:**
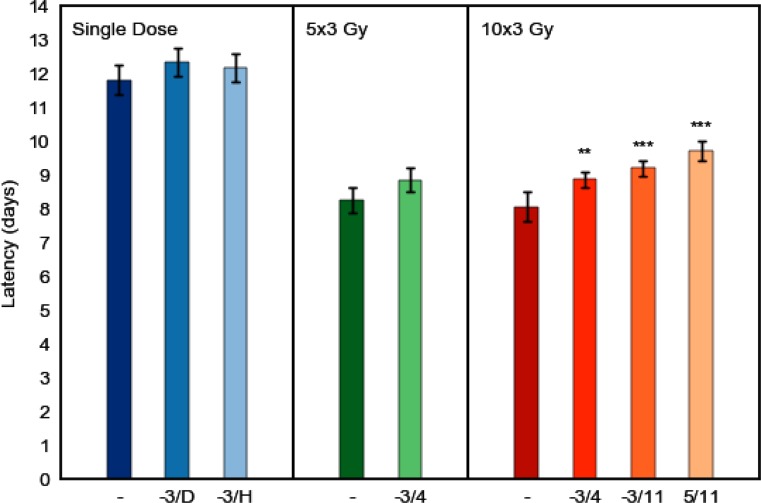
Effect of systemic DS treatment on latent times to mucosal ulceration. Irradiation protocols comprised single dose irradiation or daily fractionated irradiation over one (days 0–4) or two weeks (0–4, 7–11), followed by graded top-up doses (5 dose groups, 10 animals each) on day 7 or 14, respectively. The first and the last day of daily DS administration are indicated on the abscissa. Systemic DS treatment gradually increased the latency period. Significantly prolonged latent times were observed during two weeks of fractionation. Mean latencies were calculated from all responders in the respective experiment, independent of dose. *P*-values were calculated with the Student’s t‑test. ^*^p < 0.05; ^**^p < 0.01; ^***^p < 0.001

**Fig. 6:**
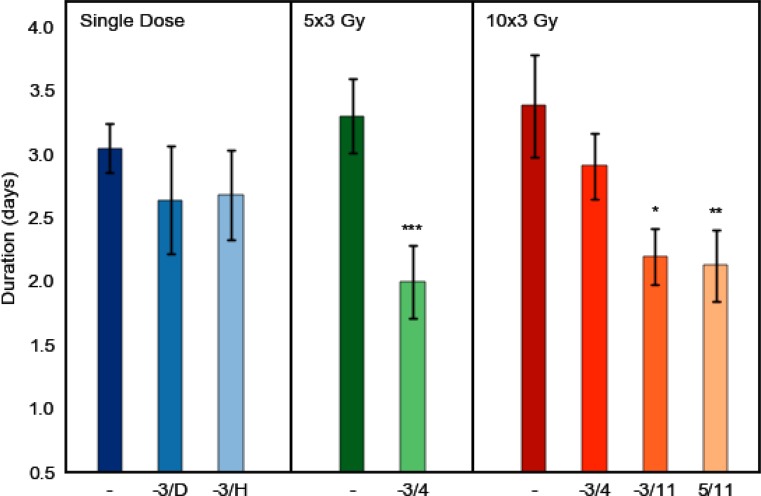
Effect of systemic DS treatment on ulcer duration. Irradiation protocols comprised single dose irradiation or daily fractionated irradiation over one (days 0–4) or two weeks (0–4, 7–11), followed by graded top-up doses (5 dose groups, 10 animals each) on day 7 or 14, respectively. The first and the last day of daily DS administration are indicated on the abscissa. Systemic DS treatment reduced the mean ulcer duration gradually with treatment time. Mean ulcer durations were calculated from all responders in the respective experiment, independent of dose. *P*-values were calculated with the Student’s t‑test. ^*^p < 0.05; ^**^p < 0.01; ^***^p < 0.001

Fractionated irradiation over one week or two weeks, without additional DS treatment, reduced the latent times to 8.2 ± 0.8 days and 8.1 ± 0.9 days, compared to single dose irradiation. The average ulcer duration was slightly prolonged to 3.3 ± 0.6 days and 3.4 ± 0.8 days, respectively. DS treatment during one week of fractionated irradiation, from days −3 to 4, prolonged the average latent time to a mean of 8.9 ± 0.7 days (*p* = 0.060) and reduced ulcer duration to 2.0 ± 0.6 days (*p* ≤ 0.001), compared to the effect of fractionation alone. When DS was applied from day −3 until day 4 of two weeks of fractionated irradiation, the latency was prolonged to 8.9 ± 0.5 days (*p* = 0.008) and ulcerations lasted for 2.9 ± 0.5 days (*p* = 0.150). DS treatment during both weeks of fractionated irradiation, from day −3 until day 11, significantly prolonged latent times to 9.2 ± 0.5 days (*p* ≤ 0.001) and accelerated healing, which left the average ulceration duration at 2.2 ± 0.5 days (*p* = 0.027). When DS administration was limited to the second week of two weeks of fractionated irradiation, latent times were prolonged to 9.7 ± 0.6 days (*p* < 0.001) and ulcer duration reduced to 2.1 ± 0.6 days (*p* = 0.002), compared to two weeks of fractionation alone (Figs. 5 and 6).

## Discussion

Oral mucositis is the most frequently occurring and dose-limiting early side effect of head-and-neck cancer radio(chemo)therapy [4, 27]. Despite a plethora of experimental approaches, so far no effective biology-based treatment has been implemented in clinical routine [8, 28].

DS exists either as soluble free glycosaminoglycan or constitutes an abundantly expressed proteoglycan, when covalently linked to a protein core. Sulfated glycans are key players in molecular and cellular events of inflammation, chemokine regulation and leukocyte guidance [18, 21], which could influence the inflammatory response in irradiated tissues [16]. Additionally, DS has a strong anticoagulatory activity [20], which may target the early onset of hypoxic conditions, which have been demonstrated in irradiated oral mucosa [17]. Furthermore, DS exerts functions in extracellular matrix assembly, fibroblast activity and wound repair processes [19, 29–31]. Therefore, DS treatment could effectively modify various radiation-induced or -altered cellular or cell-matrix interactions [32–34]. To date, clinical applications of DS are scarce, although several preclinical and clinical studies revealed an array of potential indications. Specifically, the antithrombotic properties of DS have been shown to be of clinical relevance. The studies revealed a predictable concentration-dependent dose response and overall clinical safety. To date, no DS therapy-associated bleeding complications have been reported, which makes DS a promising anticoagulation candidate for patients with bleeding disorders [35, 36].

Furthermore, its known role as FGF-10 cofactor links DS to wound-healing applications [31, 37]. No clinical experience is available so far. Recently, an in vitro study demonstrated stimulation of wound repair for a glycosaminoglycan mixture, including DS, which was based on stimulated cell migration and reduced inflammation [38]. A potential DS-mediated stimulation of cell migration, however, will have to be closely investigated with respect to a potential application during tumour therapy.

In this study, (Table 1; Fig. 4), DS treatment in combination with single dose irradiation significantly increased the isoeffective doses in both protocols tested. Moreover, DS markedly, but not significantly, reduced ulcer duration and prolonged latent times (Table 1; Figs. 5 and 6). No additional beneficial effect was found when DS administration was continued beyond the time of ulcer diagnosis.

Similar significant effects were observed with fractionated irradiation over one week as well as two weeks. DS treatment significantly increased the isoeffective doses in all fractionation protocols tested, except when DS treatment was limited exclusively to the first week of two weeks of fractionation. The most significant reduction of ulcer incidence was observed for the longest DS administration interval (day −3/11). However, DS administration over the second week of fractionation only was fully sufficient to achieve a highly significant protection of the oral mucosa, while a limitation of DS treatment to the first week of fractionation alone failed significance.

Latent times until onset of mucositis were significantly prolonged in all DS protocols tested, when given during two weeks of fractionation. However, no significant effect was observed during one week of fractionation. Ulcer duration was significantly reduced by DS treatment in all fractionation protocols, except when DS administration was limited to the first week of two weeks of fractionation.

The mucosal radiation tolerance to fractionated irradiation is mainly based on the epithelial reaction to (stem) cell depletion, i. e. repopulation, which is associated with a proliferative reorganisation within the epithelium, starting in the second treatment week and leading to increased radiation tolerance with increased treatment time [39, 40]. This highly complex procedure involves three main mechanisms, summarized as “3 A”: asymmetry loss of stem cell divisions, acceleration of the stem cell cycle and abortive divisions of lethally damaged cells [41]. According to the stem cell hypothesis, the radiosensitivity of any tissue is directly proportional to the number and the intrinsic radiosensitivity of tissue-specific stem cells, which are present within the target volume at the time of irradiation [42]. The mucositis-ameliorating activity of DS could be related to several potential mechanisms, including an increase in stem cell numbers during the pretreatment days (days −3 until −1, day 0 being the day of irradiation). This, as well as stimulation of proliferation, could lead to increased cell numbers at the time of irradiation, thus counteracting hypoplasia until repopulation becomes active. The significant increased isoeffective doses and the increased latency after DS administration in combination with single dose irradiation, as well as the failed significance after application over only the first week of two weeks of fractionated irradiation could indicate such a scenario. Augmented intercellular conjunction, e. g. through a DS-mediated reinforcement of tight and/or adherens junctions, could similarly lead to these observations, furthermore explaining the shortened ulcer duration times. Likely, other factors, such as a modulation of radiation-induced inflammation and/or perfusion could contribute to the significant mucositis-ameliorating effects of a systemic DS treatment. The mucoprotective potential of DS might be based on an interrelation of several mechanisms.

## Conclusion

In this study, DS demonstrated significant potential to ameliorate oral mucositis during single dose and conventionally fractionated irradiation. Not only the incidence of ulcerations was found to be significantly reduced, but DS additionally influenced the investigated time course parameters. Therefore, DS may be considered for clinical studies. The exact mechanisms underlying the mucositis-modulating effects of DS, however, have to be defined in further, mechanistic investigations.

## References

[1] Vera-Llonch M, Oster G, Hagiwara M, Sonis S (2006). Oral mucositis in patients undergoing radiation treatment for head and neck carcinoma. Cancer.

[2] Vissink A, Jansma J, Spijkervet FKL (2003). Oral sequelae of head and neck radiotherapy. Crit Rev Oral Biol Med.

[3] Elting LS, Cooksley CD, Chambers MS, Garden AS (2007). Risk, outcomes, and costs of radiation-induced oral mucositis among patients with head-and-neck malignancies. Int J Radiat Oncol Biol Phys.

[4] Wygoda A, Rutkowski T, Hutnik M (2013). Acute mucosal reactions in patients with head and neck cancer. Three patterns of mucositis observed during radiotherapy. Strahlenther Onkol.

[5] Herrmann T, Baumann M (2005). Prolongation of latency or overall treatment time by unplanned radiation pauses. The clinical importance of compensation. Strahlenther Onkol.

[6] Bese NS, Hendry J, Jeremic B (2007). Effects of prolongation of overall treatment time due to unplanned interruptions during radiotherapy of different tumor sites and practical methods for compensation. Int J Radiat Oncol Biol Phys.

[7] Keefe DM, Schubert MM, Elting LS (2007). Updated clinical practice guidelines for the prevention and treatment of mucositis. Cancer.

[8] Lalla RV, Bowen J, Barasch A (2014). MASCC/ISOO clinical practice guidelines for the management of mucositis secondary to cancer therapy. Cancer.

[9] Rao S, Hegde SK, Rao P (2017). Honey mitigates radiation-induced oral mucositis in head and neck cancer patients without affecting the tumor response. Foods.

[10] Ryu JK, Swann S, LeVeque F (2007). The impact of concurrent granulocyte macrophage-colony stimulating factor on radiation-induced mucositis in head and neck cancer patients: a double-blind placebo-controlled prospective phase III study by Radiation Therapy Oncology Group 9901. Int J Radiat Oncol Biol Phys.

[11] Gouvêa de Lima A, Villar RC, de Castro G (2012). Oral mucositis prevention by low-level laser therapy in head-and-neck cancer patients undergoing concurrent chemoradiotherapy: a phase III randomized study. Int J Radiat Oncol Biol Phys.

[12] Sonis ST (2007). Pathobiology of oral mucositis: novel insights and opportunities. J Support Oncol.

[13] Dörr W, Hamilton CS, Boyd T (2002). Radiation-induced changes in cellularity and proliferation in human oral mucosa. Int J Radiat Oncol Biol Phys.

[14] Gruber S, Bozsaky E, Roitinger E (2017). Early inflammatory changes in radiation-induced oral mucositis effect of pentoxifylline in a mouse model. Strahlenther Onkol.

[15] Jaal J, Richter C, Dörr W (2010). Effect of recombinant human keratinocyte growth factor (Δ23rHuKGF, Palifermin) on inflammatory and immune changes in mouse tongue during fractionated irradiation. Int J Radiat Biol.

[16] Russi EG, Raber-durlacher JE, Sonis ST (2014). Local and systemic pathogenesis and consequences of regimen-induced inflammatory responses in patients with head and neck cancer receiving Chemoradiation. Mediators Inflamm..

[17] Gruber S, Hamedinger D, Bozsaky E (2015). Local hypoxia in oral mucosa ( mouse ) during daily fractionated irradiation—effect of pentoxifylline. Radiother Oncol.

[18] Valognes D, Scheer A, Schwarz MK (2012). Glycosaminoglycan analogs as a novel anti-inflammatory strategy. Front. Immunol..

[19] Peplow PV (2005). Glycosaminoglycan : a candidate to stimulate the repair of chronic wounds. Thromb. Haemost..

[20] Trowbridge JM, Gallo RL (2002). Dermatan sulfate : new functions from an old glycosaminoglycan. Glycobiology.

[21] Belmiro CLR, Gonçalves RG, Kozlowski EO (2011). Dermatan sulfate reduces monocyte chemoattractant protein 1 and TGF-β production , as well as macrophage recruitment and myofibroblast accumulation in mice with unilateral ureteral obstruction Dermatan sulfate reduces monocyte chemoattractant protein 1 and TGF-β production , as well as macrophage recruitment and myofibroblast accumulation in mice with unilateral ureteral obstruction. Braz J Med Biol Res.

[22] Mizumoto S, Fongmoon D, Sugahara K (2013). Interaction of chondroitin sulfate and dermatan sulfate from various biological sources with heparin-binding growth factors and cytokines. Glycoconj J.

[23] Gruber S, Schmidt M, Bozsaky E (2014). Modulation of radiation-induced oral mucositis by pentoxifylline: preclinical studies. Strahlenther Onkol.

[24] Kuess P, Bozsaky E, Hopfgartner J (2014). Dosimetric challenges of small animal irradiation with a commercial X‑ray unit. Z Med Phys.

[25] Frings K, Gruber S, Kuess P (2016). Modulation of radiation-induced oral mucositis by thalidomide preclinical studies.

[26] Dörr W, Brankovic K, Hartmann B (2000). Repopulation in mouse oral mucosa: changes in the effect of dose fractionation. Int J Radiat Biol.

[27] Elting LS, Keefe DM, Sonis ST (2008). Patient-reported measurements of oral mucositis in head and neck cancer patients treated with radiotherapy with or without chemotherapy: demonstration of increased frequency, severity, resistance to palliation, and impact on quality of life. Cancer.

[28] Jensen SB, Peterson DE (2014). Oral mucosal injury caused by cancer therapies: current management and new frontiers in research. J Oral Pathol Med.

[29] Nikolovska K, Renke JK, Jungmann O (2014). A decorin-de fi cient matrix affects skin chondroitin/dermatan sulfate levels and keratinocyte function *.. Matrix Biol.

[30] Penc SF, Pomahac B, Winkler T (1998). Dermatan Sulfate Released after Injury Is a Potent Promoter of Fibroblast Growth Factor-2 Function *.. J Biol Chem.

[31] Trowbridge JM, Rudisill JA, Ron D, Gallo RL (2002). Dermatan sulfate binds and potentiates activity of keratinocyte growth factor ( FGF-7 ) *.. J Biol Chem.

[32] Somosy Z, Horvath G, Bognar G, Koteles G (2003). Structural and functional changes of cell junctions on effect of ionizing radiation. Acta Biol Szeged.

[33] Shukla PK, Gangwar R, Manda B (2016). Rapid disruption of intestinal epithelial tight junction and barrier dysfunction by ionizing radiation in mouse colon in vivo : protection by. Am J Physiol Gastrointest Liver Physiol.

[34] Wardill HR, Logan RM, Bowen JM (2016). Tight junction defects are seen in the buccal mucosa of patients receiving standard dose chemotherapy for cancer. Support Care Cancer.

[35] Cofrancesco E, Boschetti C, Gianese F, Cortellaro M (1994). Dermatan sulfate for the treatment of disseminated Intravascular coagulation (DIC) in acute leukaemia: a randomised heparin-controlled pilot study. Thromb Res.

[36] Gabella P, Ordine O, Ordine O (2012). Dermatan sulfate : an alternative to unfractionated heparin for anticoagulation in hemodialysis patients Dermatan sulfate : an alternative to unfractionated heparin for anticoagulation in hemodialysis patients. J Nephrol.

[37] Plichta J, Radek K (2013). Sugar-coating wound repair: a review of FGF-10 and dermatan sulfate in wound healing and their potential application in burn wounds. J Burn Care Res.

[38] Belvedere R, Bizzarro V, Parente L, Petrella F (2017). The pharmaceutical device Prisma ® skin promotes in vitro angiogenesis through endothelial to mesenchymal transition during skin wound healing. Int J Mol Sci.

[39] Dörr W, Emmendörfer H, Weber-Frisch M (1996). Tissue kinetics in mouse tongue mucosa during daily fractionated radiotherapy. Cell Prolif.

[40] Dorr W, Emmendorfer H, Haide E, Kummermehr J (1994). Proliferation equivalent of ‘accelerated repopulation’ in mouse oral mucosa. Int J Radiat Biol.

[41] Dörr W (1997). Three A’s of repopulation during fractionated irradiation of squamous epithelia: asymmetry loss, acceleration of stem-cell divisions and abortive divisions. Int J Radiat Biol.

[42] Dörr W (2015). Radiobiology of tissue reactions. Ann ICRP.

